# Hearing loss: an unusual presentation of neurobrucellosis: a case report

**DOI:** 10.1186/s13256-023-03836-x

**Published:** 2023-03-29

**Authors:** Prem Kumar Sah, Hari Krishna Lamichhane, Ezza Fatima Tariq, K. C. Saroj, Prabhat Adhikari

**Affiliations:** 1 Manipal Teaching Hospital, Pokhara, Nepal; 2 Multan Medical and Dental College, Multan, Pakistan; 3 Institute of Medicine, Maharajgunj, Kathmandu Nepal

**Keywords:** Neurobrucellosis, Brucellosis, Nepal, Hearing loss, Sensorineural hearing loss, Case report, Brucella agglutination test, Unusual presentation, Zoonotic disease

## Abstract

**Introduction:**

Brucellosis is a zoonotic disease, caused by a Gram-negative coccobacillus of *Brucella* genus, transmitted to humans by animals, especially cattle. It rarely involves the nervous system (neurobrucellosis); only a few cases present with hearing loss. We report a case of neurobrucellosis, that presented with bilateral sensorineural hearing loss and mild to moderate persistent headache. To the best of our knowledge, this is the first well-documented case from Nepal.

**Case presentation:**

The patient was a 40-year-old Asian male shepherd from the western mountainous region of Nepal who came to the emergency department of Manipal Teaching Hospital, Pokhara in May, 2018 and did a follow-up for 6 months. He presented with high-grade fever, profuse sweating, headache, myalgia, and bilateral sensorineural hearing loss. His history of consuming raw milk of cattle, symptoms including persistent mild to moderate headache, bilateral hearing loss, and serological findings were suggestive of neurobrucellosis. Following treatment, the symptoms improved, including the complete recovery of hearing loss.

**Conclusion:**

Hearing loss may be the manifestation of neurobrucellosis. Physicians should know about such presentations in brucella endemic areas.

## Background

Brucellosis is a chronic granulomatous disease that usually presents with vague symptoms such as fever, diaphoresis, malaise, myalgia, arthralgia, and headache, as well as some atypical symptoms because of multiple organ involvement [1, 2]. Brucella is an intracellular bacterium, and has a predilection for reticuloendothelial system involvement, and can present as splenomegaly (34%), hepatomegaly (8.6%), and lymphadenopathy (10.7%) [2, 3]. Hematological abnormalities include mild anemia, leukopenia, thrombocytopenia, or pancytopenia. The likely causes of pancytopenia in brucellosis are hypersplenism, autoimmune destruction, bone marrow granuloma, and bone marrow suppression [2, 4].

Neurobrucellosis is seen in up to 10% of cases of brucellosis [5–7]. Neurobrucellosis has diverse presentations: meningitis, encephalitis, brain abscess, subarachnoid hemorrhage, neuropathies (cranial and peripheral), and psychiatric manifestations [1, 8]. Thus, many of these patients land up in neurology and otolaryngology clinics. Hearing loss may be an important manifestation of neurobrucellosis in brucella endemic areas. The vestibulocochlear nerve seems to be the most commonly affected cranial nerve (10%) [9]. We report a case of a 40-year-old man with neurobrucellosis, where bilateral sensorineural hearing loss, persistent mild to moderate headache, and positive cerebrospinal fluid (CSF) brucella agglutination test with significant antibody titer (1/256) were present.

## Case presentation

**Fig. 1:**
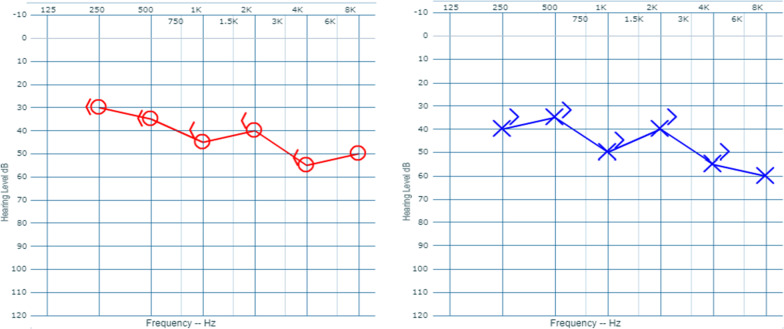
—air conduction right ear;  —bone conduction right ear,  —air conduction left ear;  —bone conduction left ear

We report a case of a 40-year-old Asian male shepherd who presented with the chief complaint of recurrent high-grade fever with an evening rise in temperature of 1 month duration associated with profuse sweating, mild to moderate headache, myalgia, and anorexia. He had experienced decreased hearing in both ears for the last 10 days. He noticed his clothes became too loose for him. He did not report diabetes mellitus, hypertension, or any other chronic illness. The patient had a high fever with a 103° F recorded temperature, tachycardia with a pulse rate of 110/min, low body mass index (BMI) of 18, normal blood pressure, and a respiratory rate of 18/min. Physical examination revealed splenomegaly (14 cm), but no hepatomegaly or enlarged lymph nodes. Whisper, Rinne, and Weber tests were performed and sensorineural hearing loss was suggested by deafness to whispering sound, air conduction better than bone conduction, and no lateralization. Pure tone audiometry showed bilateral mild-moderate sensorineural hearing loss (Fig. 1).

**Fig. 2:**
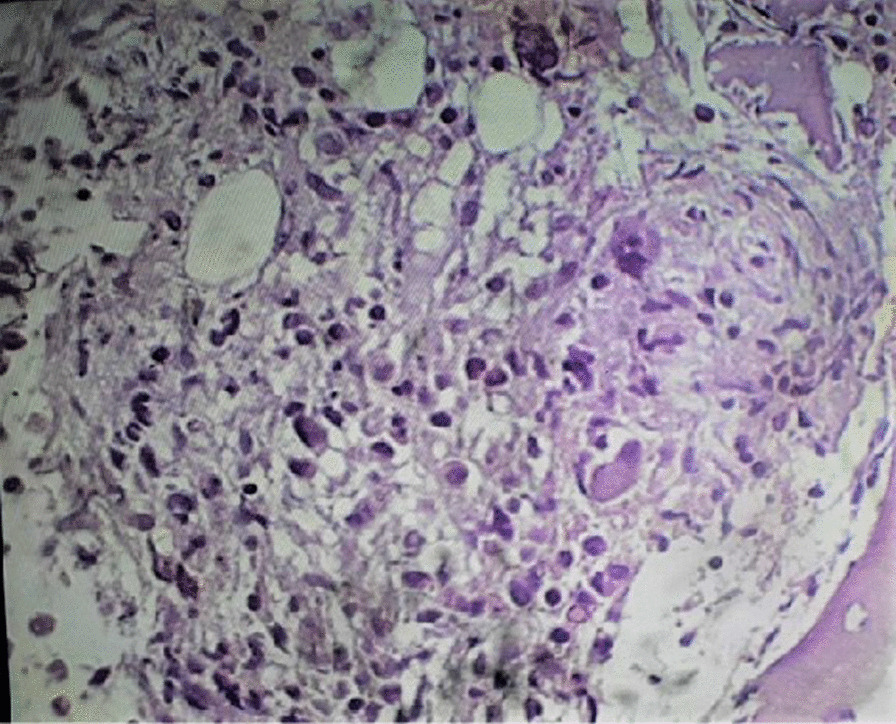
Bone marrow biopsy: non-caseating granuloma

Our initial investigation showed pancytopenia with elevated erythrocyte sedimentation rate (ESR) (34 mm in 1st hour). Tuberculin skin test for tuberculosis, K39 serology for leishmaniasis, widal test for typhoid, thick and thin smear for plasmodium were performed and all test results were negative. Bone marrow aspiration cytology and trephine biopsy with H&E stain showed reactive marrow with non-caseating granulomatous inflammation (Fig. 2). After taking in-depth history again, we found that he regularly consumed raw milk from goats and sheep on his farm. We suspected neurobrucellosis and performed a lumbar puncture that showed normal opening pressure and normal CSF findings including glucose, protein, and leukocytes. However, the Brucella agglutination test in blood and CSF came positive with antibody titer of 1/256 in both. Acid Fast Bacilli smear, treponema pallidum hemagglutination gram stain, fungal stain and bacterial culture of CSF were all negative.

Considering the consumption of cattle’s raw milk, endemicity of brucella in Nepal, neurological findings unexplainable otherwise, and positive CSF brucella agglutination test, we diagnosed the case as neurobrucellosis. We treated him with doxycycline (100 mg twice a day for 6 months), and streptomycin injections (1 g/day for 21 days). Follow-up after three months showed complete resolution of symptoms, except hearing loss that improved after completion of therapy at 6 months. To the best of our knowledge, this is the first case report of neurobrucellosis from Nepal.

## Discussion and conclusions

Brucellosis has a wide geographical distribution with over 500,000 human cases per year worldwide [1, 10, 11]. The prevalence of brucellosis among cattle has been well-known in Nepal, however, there is very little evidence of human brucellosis. An animal study in Nepal showed Brucellosis seropositivity rate to be 12% [12]. Therefore, the traditional practice of consuming raw milk and its products, eating raw meat from cattle, and cattle rearing as an occupation, pose a serious threat of human brucellosis in this country [12, 13].

Although there are no definite criteria for Neurobrucellosis [14], some articles have also mentioned that neurobrucellosis can be diagnosed by any of the following criteria: signs and symptoms consistent with neurobrucellosis, isolation of brucella from CSF and/or positive brucella agglutination titer in CSF, presence of lymphocytosis, increased protein, and decreased glucose levels in CSF or diagnostic findings in cranial magnetic resonance imaging or computed tomography (MRI or CT) [8, 15]. Neurobrucellosis (NB) has neither a typical clinical picture nor specific cerebrospinal fluid (CSF) findings. Imaging findings of neurobrucellosis can be divided into four categories: normal, inflammatory (evident by granulomas and enhancement of meninges, perivascular space, or lumbar nerve roots), changes in white matter, and vascular changes [16]. The parenchymal nerve and glial cells are not infected with brucella and the inflammatory reaction in the brain is limited to the meninges and the blood vessels [17]. We suspected neurobrucellosis because of clinical presentation, geographical co-relation, the significant titer of brucella agglutination test in CSF & blood, resolution of symptoms after treatment, and exclusion of other common etiologies. Our patient did not have a history of head trauma, prior use of ototoxic drugs, or exposure to loud noise, so we ruled out common causes of hearing loss on clinical grounds. We found an improvement in hearing with treatment which strongly suggest that hearing loss could be due to brucella infection. Limited literatures are available that suggests inner ear damage is caused by brucella endotoxin penetrating the labyrinth, causing microvascular spasms and thus affecting the cochlear nerve or involving the central auditory pathway [18–20]. Early diagnosis and treatment may completely resolve hearing loss [18].

There is no consensus on the dose, type of antibiotics, and duration of treatment in Neurobrucellosis [15, 21, 22]. Dual or triple antibiotic combination of doxycycline, Trimethoprim-Sulfamethoxazole (TMP-SMX), streptomycin, rifampicin, or ceftriaxone for > 2 months (3–6 months) has been recommended in various studies. The total duration of treatment may be extended beyond 6 months depending upon the individual patient, clinical assessment, CSF findings, and radiological findings [15, 23–25]. Doxycycline is the drug of choice in Neurobrucellosis as it has better CNS penetration, and a long half-life [15]. Treatment response can be determined by the improvement in clinical, and lab parameters [22]. We treated the patient with a combination of Doxycycline and Streptomycin, instead of Rifampicin, as it is the reserved drug for tuberculosis in our country.

Brucellosis is often misdiagnosed as malaria, tuberculosis, and typhoid fever which may cause mistreatment and under-reporting in Nepal. Our case met the diagnostic criteria of Neurobrucellosis; therefore, this is the first case reported from Nepal, with sensorineural hearing loss and headache as a manifestation of the disease. Knowing this rare possibility, clinicians should include brucella tests in patients presenting with hearing loss, in brucella endemic regions as early diagnosis and treatment have better outcomes. This case should affect the practice of physicians who interact with sensorineural hearing loss in brucella endemic areas.

Since Brucellosis is a zoonotic disease, we suggest clinicians collaborate with veterinarians who are involved in identification and treatment of infected animals, as this will help prevent further transmission to humans. This is also the core of the “One Health Policy” by World Health Organization (WHO) in controlling zoonotic diseases [26]. The government should expand the testing capacity for Brucellosis all over Nepal and prioritize this infectious disease. Similarly, medics and researchers from Nepal should focus on the incidence and prevalence of this disease to know the burden of human brucellosis in Nepal.

## Limitations of the study

Neuroimaging could not be performed due to a shortage of economic resources.

## Data Availability

All data generated or analyzed during this study are included in this published article.

## Abbreviations

BMI: Body mass index
H&E: Hematoxylin and eosin
ESR: Erythrocyte sedimentation rate
CSF: Cerebrospinal fluid
WHO: World health organization
TMP-SMX: Trimethoprim and sulfamethoxazole

## References

[1] Pappas G, Papadimitriou P, Akritidis N, Christou L, Tsianos EV (2006). The new global map of human brucellosis. Lancet Infect Dis.

[2] Jiang W, Chen J, Li Q, Jiang L, Huang Y, Lan Y (2019). Epidemiological characteristics, clinical manifestations and laboratory findings in 850 patients with brucellosis in Heilongjiang Province, China. BMC Infect Dis.

[3] Dreshaj S, Shala N, Dreshaj G, Ramadani N, Ponosheci A (2016). Clinical manifestations in 82 neurobrucellosis patients from Kosovo. Mater Sociomed.

[4] Hamilton PK (1954). The bone marrow in brucellosis. Am J Clin Pathol.

[5] Ucmak H, Kokoglu O, EKPJ of, 2012 undefined. Hearing loss and accompaning disturbance of Neurobrucellosis cases. researchgate.net. https://www.researchgate.net/profile/Hasan-Ucmak/publication/237303488_Hearing_loss_and_accompaning_disturbance_of_neurobrucellosis_cases/links/0deec51bb1dec50ce0000000/Hearing-loss-and-accompaning-disturbance-of-neurobrucellosis-cases.pdf

[6] Araj GF (2010). Update on laboratory diagnosis of human brucellosis. Int J Antimicrob Agents.

[7] Buzgan T, Karahocagil MK, Irmak H, Baran AI, Karsen H, Evirgen O (2010). Clinical manifestations and complications in 1028 cases of brucellosis: a retrospective evaluation and review of the literature. Int J Infect Dis.

[8] McLean DR, Russell N, Khan MY (1992). Neurobrucellosis: clinical and therapeutic features. Clin Infect Dis.

[9] Bajin MD, Savaş Ö, Aslan F, Sennaroğlu L (2016). Cochlear implantation in neurobrucellosis. Balkan Med J.

[10] Alkahtani AM, Assiry MM, Chandramoorthy HC, Al-Hakami AM, Hamid ME (2020). Sero-prevalence and risk factors of brucellosis among suspected febrile patients attending a referral hospital in southern Saudi Arabia (2014–2018). BMC Infect Dis.

[11] Bosilkovski M, Krteva L, Dimzova M, Kondova I (2007). Brucellosis in 418 patients from the Balkan Peninsula: exposure-related differences in clinical manifestations, laboratory test results, and therapy outcome. Int J Infect Dis.

[12] Pandeya Y, Joshi D, Dhakal S, Ghimire L, Mahato B, Chaulagain S (2013). Seroprevalence of brucellosis in different animal species of Kailali district, Nepal. Int J Infect Microbiol.

[13] Gompo TR, Shah R, Tiwari I, Gurung YB (2021). Sero-epidemiology and associated risk factors of brucellosis among sheep and goat population in the south western Nepal: a comparative study. BMC Vet Res.

[14] Raina S, Sharma A, Sharma R, Bhardwaj A (2016). Neurobrucellosis: a case report from Himachal Pradesh, India, and review of the literature. Case Rep Infect Dis.

[15] Guven T, Ugurlu K, Ergonul O, Celikbas AK, Gok SE, Comoglu S (2013). Neurobrucellosis: clinical and diagnostic features. Clin Infect Dis.

[16] Kizilkilic O, Calli C (2011). Neurobrucellosis. Neuroimaging Clin N Am.

[17] Moreno E, Barquero-Calvo E (2020). The role of neutrophils in brucellosis. Microbiol Mol Biol Rev.

[18] Kaygusuz TO, Kaygusuz I, Kilic SS, Yalcin S, Felek S (2005). Investigation of hearing loss in patients with acute brucellosis by standard and high-frequency audiometry. Clin Microbiol Infect.

[19] Bayazit YA, Namiduru M, Bayazit N, Özer E, Kanlikama M (2002). Hearing status in brucellosis. Otolaryngol Head Neck Surg.

[20] Gul HC, Erdem H, Bek S (2009). Overview of neurobrucellosis: a pooled analysis of 187 cases. Int J Infect Dis.

[21] Aktürk H, Özkan A, Odabaşı İ, Uzunhan T, Gürler N, Erol O (2015). Hearing loss: can it be neurobrucellosis?. J Microbiol Infect Dis.

[22] Ceran N, Turkoglu R, Erdem I, Inan A, Engin D, Tireli H (2011). Neurobrucellosis: clinical, diagnostic, therapeutic features and outcome. Unusual clinical presentations in an endemic region. Braz J Infect Dis.

[23] Zhao S, Cheng Y, Liao Y, Zhang Z, Yin X, Shi S (2016). Treatment efficacy and risk factors of neurobrucellosis. Med Sci Monit.

[24] Cem Gul H, Erdem H, Gorenek L, Fatih Ozdag M, Kalpakci Y, Yasar Avci I, et al. Management of neurobrucellosis: an assessment of 11 cases. jstage.jst.go.jp. https://www.jstage.jst.go.jp/article/internalmedicine/47/11/47_11_995/_article/-char/ja/10.2169/internalmedicine.47.0866 18520109

[25] Pappas G, Akritidis N, Christou L (2007). Treatment of neurobrucellosis: what is known and what remains to be answered. Expert Rev Anti Infect Ther.

[26] Hitziger M, Esposito R, Canali M, Aragrande M, Häsler B, Rüegg SR (2018). Knowledge integration in one health policy formulation, implementation and evaluation. Bull World Health Organ.

